# Emotional Responses to Social Media and Non‐Suicidal Self‐Injury Among Adolescents

**DOI:** 10.1111/sltb.70122

**Published:** 2026-06-25

**Authors:** Athena B. Thai, Melissa J. Dreier, Jessica L. Hamilton

**Affiliations:** ^1^ Department of Psychology Rutgers University Piscataway New Jersey USA

**Keywords:** adolescents, non‐suicidal self‐injury, social media

## Abstract

**Introduction:**

Adolescents engage in non‐suicidal self‐injury (NSSI) and social media more than any other age group. To understand the role of adolescents' subjective social media experiences in NSSI, this study examined the relationships between emotional responses to social media and NSSI urges and engagement.

**Methods:**

Data were collected from two studies, which included a cross‐sectional study (*N* = 408) and an 8‐week intensive‐longitudinal study (*N* = 80) with ecological momentary assessment (3 surveys/day).

**Results:**

The cross‐sectional study revealed that positive responses to social media were associated with the presence of NSSI urges across all time points and frequency of NSSI engagement in the past year and lifetime. Negative responses to social media were associated with the presence of NSSI urges and engagement across all time points and frequency of NSSI engagement in the past week, month, and year. The intensive‐longitudinal study revealed that positive responses to social media were not associated with daily NSSI urge intensity or presence of engagement, although negative responses to social media were associated with both daily NSSI urge intensity and presence of engagement.

**Discussion:**

Interventions aiming to reduce adolescent NSSI may benefit from addressing ways to cope with negative social media experiences and promoting positive experiences.

## 1 Introduction

### 1.1 Non‐Suicidal Self‐Injury

Non‐suicidal self‐injury (NSSI), the deliberate harm to one's body without suicidal intent (Klonsky et al. 2014), is a known predictor for subsequent suicide attempts (Mars et al. 2019). In adolescents, the lifetime prevalence of NSSI is 22%, with 19% in the past year (Xiao et al. 2022; Gillies et al. 2018; Farkas et al. 2024). Thoughts about NSSI–including general thoughts, plans of engagement, and mental imagery of NSSI–are one of the most prominent predictors of NSSI engagement (Andrewes et al. 2017). In particular, the urge to engage in NSSI is a specific type of NSSI thought characterized by a strong desire to self‐harm without suicidal intent. NSSI urges have been identified as a predictor of both proximal and long‐term NSSI engagement, with more intense NSSI urges predicting a higher likelihood of NSSI engagement (Burke et al. 2022; Fitzpatrick et al. 2020). Researching factors associated with NSSI urges and urge intensity in addition to NSSI engagement may contribute to targeted interventions (Burke et al. 2022). Given that NSSI is a strong predictor of the transition from suicidal thoughts to suicide attempts, interventions targeting NSSI may also reduce the risk of suicidal behavior (Horvath et al. 2020).

Experiencing negative life events and interpersonal stressors has been shown to precede NSSI urges and engagement (Turner, Cobb, et al. 2016; Turner, Yiu, et al. 2016; Victor et al. 2018), as adolescents may engage in NSSI to decrease emotional distress in response to these negative events (Liu et al. 2016; Baetens et al. 2021). Research utilizing ecological momentary assessment (EMA) has also identified how individuals' emotional responses to difficult events may be linked to NSSI in real time. For instance, experiencing greater‐than‐usual negative emotional reactions to daily social stressors is associated with same‐day NSSI urges and behaviors (Haliczer and Dixon‐Gordon 2023). Individuals have also reported experiencing a more intense negative mood on days they engaged in NSSI in comparison to days they did not (Turner, Cobb, et al. 2016; Turner, Yiu, et al. 2016). Thus, both negative experiences and emotions are associated with NSSI, and negative experiences may be linked to NSSI through their influence on individuals' emotions (Jiang et al. 2022).

### 1.2 Social Media

As there has been a simultaneous increase in social media use and adolescent NSSI, research has begun to explore the relationship between these constructs (Spinola et al. 2024), such that greater time spent on social media is associated with increased NSSI in adolescents (Memon et al. 2018).

Emerging research indicates that specific experiences on social media and emotional responses to those experiences may be more specifically linked to mental health outcomes, rather than screen time (Nesi et al. 2022). For instance, viewing self‐harm content on social media is associated with weekly NSSI urges and behaviors, such that exposure to self‐harm content may be a proximal risk factor for NSSI (Hamilton, Dalack, et al. 2024; Hamilton, Untawale, et al. 2024). Hamilton, Dalack, et al. (2024) and Hamilton, Untawale, et al. (2024) also found that positive social media experiences are associated with a lower likelihood of suicidal ideation, whereas negative social media experiences are associated with a higher likelihood of suicidal ideation. Experiencing a greater‐than‐usual positive mood on social media is also associated with a lower likelihood of experiencing suicidal thoughts that same day (Dreier et al. 2025). However, specific activities online, such as the number of pictures posted or comments received, are not associated with acute suicidality in individuals with past NSSI (Brown et al. 2019). Thus, it is possible that individual differences between adolescents' social media use and subsequent emotional responses may account for these mental health outcomes (Valkenberg and Peter 2013), as expression of negative emotions on social media is associated with acute suicidality (Brown et al. 2019). It may be that subjective emotional experiences are the mechanism through which NSSI manifests, rather than social media features or use. This may be because negative experiences on social media are associated with sustained negative affect (Boyd et al. 2024). It is therefore possible that when adolescents are exposed to negative content or experiences online, they experience more negative affect, which may increase their risk for NSSI.

As social media becomes an increasingly prevalent space for adolescents' experiences, certain features of social media also create new types of positive experiences (Nesi et al. 2018). There is potential for harm through social comparison online, but also potential for benefits through social support (Marchant et al. 2018). For instance, positive social media experiences may buffer against self‐injury when used for distraction or support, as reported by many youth following recent suicide attempts (Nesi et al. 2019). Many adolescents report benefits from using social media through accessing social connection and by seeking humorous content that enhances positive affect (Weinstein et al. 2021). This is further supported by Hamilton, Dalack, et al. (2024) and Hamilton, Untawale, et al. (2024), which demonstrates that on days when adolescents felt more supported on social media, positive social media experiences were associated with lower likelihood of suicidal ideation, whereas negative experiences were associated with higher likelihood of suicidal ideation. Connectedness is similarly a protective factor for adolescent NSSI (Shi et al. 2025), so it is possible that positive emotional responses to social media through online peer connection may reduce the likelihood of NSSI.

### 1.3 The Current Study

Adolescents both engage in social media (Shimoga et al. 2019) and NSSI more than any age group (Swannell et al. 2014). Current research on social media and mental health outcomes which rely on cross‐sectional data may be beneficial in identifying associations and may yield insight into broader patterns, but may not capture the daily fluctuations in social media experiences or NSSI (Goreis et al. 2025). Intensive‐longitudinal designs may be better suited to capturing these fluctuations and examining individuals' experiences in real‐time (Hamilton, Dalack, et al. 2024; Hamilton, Untawale, et al. 2024). Previous research examining social media and NSSI using intensive‐longitudinal designs has identified an association between viewing self‐harm content exposure online and weekly NSSI urges and behaviors (Hamilton, Dalack, et al. 2024; Hamilton, Untawale, et al. 2024), and among adolescents who engage in NSSI, experiencing negative events online is also associated with increased daily stress, negative affect, and NSSI urges (Goreis et al. 2025). That being said, to our knowledge, no studies have yet explored how emotional responses to social media experiences are associated with NSSI. As positive experiences do not appear to buffer against the harmful effects of negative social media experiences on suicidal ideation (Hamilton, Dalack, et al. 2024; Hamilton, Untawale, et al. 2024), examining both positive and negative emotional responses to social media independently may be important in mental health outcomes. Given that positive and negative emotions may predict psychological outcomes through independent pathways (Ong and Ram 2016), the current study examined positive and negative emotional responses as separate constructs. Understanding this relationship will provide insight into how subjective emotional responses to social media influences NSSI, ultimately informing targeted interventions.

The current study utilized a two‐study approach to investigate the relationship between emotional responses to social media and NSSI with both a cross‐sectional and intensive longitudinal (daily level) component. By utilizing an intensive‐longitudinal design, the study is also able to identify within‐person fluctuations in NSSI and social media, allowing us to analyze changes within individuals, as both social media use and NSSI may vary from day to day. The first part of this study (Study 1) examined how positive and negative emotional responses to social media are associated with NSSI urges and engagement (including presence/absence and frequency) on a cross‐sectional self‐report survey. The second part of the study (Study 2) utilized ecological momentary assessment (EMA) to examine how greater‐than‐usual positive and negative emotional responses to social media are associated with the intensity of NSSI urges and the presence/absence of engagement at the daily level.

As the two approaches contained different measures, Study 1 measured NSSI urge through a dichotomous variable reflecting presence/absence while Study 2 measured NSSI urge intensity on a continuous scale, limiting direct comparisons of NSSI urges. However, measuring both presence/absence and urge intensity may expand our understanding of these relationships. Similarly, measurement of frequency of NSSI engagement was not available in Study 2. Although this limited our capacity to directly compare NSSI engagement frequency at the cross‐sectional versus intensive longitudinal levels, the measurement of relationships between emotional responses to social media and NSSI engagement (presence/absence) in both studies allowed direct comparison between these findings at different levels of analysis. Additional measurement of NSSI frequency (number of times an individual engaged in NSSI) in Study 1 adds additional nuance, allowing for a more comprehensive understanding of these relationships.

It was hypothesized that positive responses to social media would be associated with a lower probability of experiencing NSSI urges and engagement (presence/absence and frequency), while negative responses to social media would be associated with a greater probability of experiencing NSSI urges and engagement at the cross‐sectional level. At the daily level, it was hypothesized that positive responses to social media would be associated with less intense NSSI urges and a greater probability of engagement, while negative responses to social media would be associated with more intense NSSI urges and a lower probability of engagement.

## 2 Study 1: Cross‐Sectional Examination

### 2.1 Methods

#### 2.1.1 Participants

Participants were recruited via advertisements on Instagram and completed an online nationwide cross‐sectional survey. Eligibility criteria included being ages 13–17, living in the US, speaking English fluently, and using social media at least three times a week. Eligible participants completed an online assent form on Qualtrics and were automatically invited to complete the survey. Compensation was provided through Amazon gift cards.

Several approaches were taken to ensure high‐quality participant responses and prevent bot responses. To ensure participants were from the US, Qualtrics used IP address tracking to approximate participant location. The survey included several validation questions (e.g., “Select “moderately” in response to this question”), of which participants were required to have 85% accuracy. QreCAPTCHA scores from Qualtrics were used to determine likelihood of bot responses, with scores closer to 0 indicating a higher chance of a bot response and scores closer to 1 indicating a higher chance of a human response. Responses with a score lower than 0.5 were excluded. Participants also answered one of five qualitative questions (e.g., “What do you least like about social media?”) and responses were reviewed by undergraduate research assistants. Answers with irrelevant information, major grammatical or syntax errors (e.g., “Too much talks”, “Some have got bad influence from the social media”), and generic content (“Movies”, “Advice”) were flagged. In these cases, participants were given the opportunity to meet with the study team to ensure eligibility, which did not occur for any flagged responses.

Of the 1243 participants completed survey measures, 791 (64%) passed validation checks embedded in the survey, 418 (34%) passed qualitative response checks, and 408 (33%) fully completed all relevant study measures. Thus, a total of 408 adolescents (45% Female, 56% White) were included in the present study. Because missingness was minimal–only 2.4% of participants were excluded due to missingness–missing data was not imputed. Although systematically missing data may introduce bias, the minimal loss of data suggests that any bias is likely limited.

This study was approved by the University Institutional Review Board.

#### 2.1.2 Measures

*Emotional Responses to Social Media*: The Emotional Responses to Social Media scale contains 24 items scored on a 5‐point likert scale ranging from 0 (Never) to 4 (Always), adapted based on focus groups and youth advisory board input from a shorter version used in prior research (Nesi et al. 2022). The measure consists of two subscales. The positive subscale contains 10 items (range 0–40) measuring the frequency of positive emotional responses to experiences on social media (e.g., “When you use social media, how often do you feel proud of yourself because of something you expressed, posted, or shared?”). The negative subscale contains 14 items (range 0–56) measuring the frequency of negative emotional responses to experiences on social media (e.g., “When you use social media, how often do you feel left out or excluded by friends or people you know?”). Both subscales have had high reliability (Nesi et al. 2022). In the current study, internal consistency was high for both the positive (ɑ = 0.83) and negative (ɑ = 0.90) subscales. The full items are detailed in the Supporting Information S1.

*Self‐Injurious Thoughts and Behaviors Interview‐Revised*: To obtain data about NSSI, participants completed the 53‐item Self‐Injurious Thoughts and Behaviors Interview‐Revised, which measures the frequency and characteristics of suicide and self‐injury (Fox et al. 2020). The measure has been shown to have high convergent validity and inter‐rater reliability (Gratch et al. 2022). For the purposes of this manuscript, suicidal thoughts and suicide attempts were not included as outcome variables, since the objective of the current study was to focus on NSSI urges and engagement solely. Thus, select relevant items regarding NSSI were included in the current study.

*NSSI Urges (presence/absence)*: To assess whether participants experienced NSSI urges, 4 items were included and measured the presence of NSSI urges in the past week, month, year, and across one's lifetime, with dichotomized responses (0 or 1).

*NSSI Presence (presence/absence)*: To assess whether participants engaged in NSSI, 4 items measured the presence of NSSI engagement in the past week, month, year, and across one's lifetime, with dichotomized responses (0 or 1).

*NSSI Engagement (frequency)*: To measure the frequency of NSSI engagement, 4 items assessed how often participants engaged in NSSI across one's lifetime (0–100+ times), past year (0–100+ times), past month (0–50+ times), and in the past week (0–11 times). Participants were only shown these items if they endorsed NSSI presence previously.

*Demographics*: Participants indicated their age, gender identity, and racial/ethnic identity.

#### 2.1.3 Analytic Plan

Logistic regressions were conducted to identify associations between positive and negative emotional responses to social media and the presence of NSSI urges and engagement across the past week, month, year, and lifetime. Linear regressions were further used to examine the relationship between positive and negative emotional responses to social media and the frequency of NSSI engagement among individuals who had endorsed experiencing lifetime NSSI.

### 2.2 Results

The study contained a diverse sample. A total of 51 out of 408 (13%) of participants identified as a gender other than cisgender girl or boy, and 184 (44%) held a racial or ethnic minority identity. See Table 1 for complete demographic breakdown.

**TABLE 1:** Demographic information for Study 1.

	Response, *N* (%) or mean (SD) (*N* = 408)
Age	16.0 (0.9)
Race	
White	227 (55.6%)
Black	66 (16.2%)
Multiracial	34 (8.3%)
Asian	41 (10.0%)
Hispanic	23 (5.6%)
American Indian	15 (3.7%)
Middle Eastern	1 (0.2%)
NHPI	1 (0.2%)
Sex	
Female	230 (56.4%)
Male	178 (43.6%)
Gender	
Another gender	51 (12.5%)
Boy	173 (42.4%)
Girl	184 (45.1%)
Transgender	
Yes	139 (34.1%)
No	269 (65.9%)
Sex orientation	
LGBQ+	149 (36.5%)
Heterosexual	256 (62.7%)
Missing	3 (0.7%)

*Note:* LGBQ+ = acronym encompassing any participants whose sexual orientation is not heterosexual (e.g., gay, lesbian, bisexual).

Of the 408 participants, 137 (34%) endorsed lifetime NSSI urges, with 126 (31%) experiencing NSSI urges in the past year, 94 (23%) in the past month, and 75 (18%) in the past week. Among participants who had experienced NSSI urges, the mean age of onset for experiencing urges was age 11.74 (SD = 2.79). On average, participants endorsed experiencing NSSI urges 1.31 days per week (SD = 2.04) and 6 days per month (SD = 9.30). A total of 121 participants (30%) endorsed lifetime NSSI engagement and were thus shown items assessing frequency of NSSI engagement, with 95 endorsing engagement (23%) in the past year, 61 (15%) in the past month, and 38 (9%) in the past week. Among participants who had endorsed NSSI (*N* = 121; 30%), the mean age of onset for experiencing urges was age 11.92 (SD = 2.68). The most commonly endorsed method was cutting or carving skin (*N* = 38; 9%). Participants endorsed engaging in NSSI, on average, 0.54 times per day (SD = 1.49), 1.23 times per week (SD = 2.08), and 5.02 times per month (SD = 5.09).

Average positive ERSM score was 26 (SD: 5.91), with a range from 7 to 40. Average negative ERSM score was 29 (SD: 10.36), with a range from 0 to 55. Positive and negative ERSM scores were negatively correlated (*r* = −0.17, *p* = 0.035).

#### 2.2.1 ERSM and NSSI Primary Results

Higher positive ERSM scores were associated with a lower probability of NSSI urge presence across the lifetime, past year, past month, and past week time frames (Table 2).

**TABLE 2:** Results from logistic regression models testing associations between positive emotional responses to social media and presence of NSSI urges.

Predictors	Past week NSSI urge (presence)			Past month NSSI urge (presence)			Past year NSSI urge (presence)			Lifetime NSSI urge (presence)		
	Odds ratios	CI	*p*	Odds ratios	CI	*p*	Odds ratios	CI	*p*	Odds ratios	CI	*p*
(Intercept)	1.11	0.37–3.36	0.853	1.59	0.57–4.50	0.378	2.07	0.80–5.46	0.135	1.7	0.67–4.35	0.266
ERSM positive	0.94	0.90–0.98	**0.005**	0.94	0.90–0.97	**0.001**	0.94	0.91–0.98	**0.001**	0.95	0.92–0.99	**0.01**
Observations	408			408			408			408		
*R* ^2^ Tjur	0.019			0.025			0.025			0.016		

*Note:* Bold values indicate *p* 〈 0.05 for all tables.

Abbreviation: ERSM positive, positive emotional responses to social media.

Higher positive ERSM was not associated with lower probability of NSSI engagement presence across one's lifetime (*z* = −1.85, *p* = 0.06), nor in the past year (*z* = −1.96, *p* = 0.05, OR = 0.96), past month (*z* = −1.07, *p* = 0.28), or past week (*z* = −0.54, *p* = 0.59). Because associations between positive ERSM and NSSI engagement presence were nonsignificant across all assessed timeframes, these model results are reported in text rather than in a separate table. Higher positive ERSM scores were also not associated with frequency of past month or past week NSSI engagement (Table 3). However, higher positive ERSM scores were associated with lower frequency of lifetime NSSI engagement and past year NSSI engagement (Table 3).

**TABLE 3:** Results from linear regression models testing associations between positive emotional responses to social media and frequency of NSSI engagement.

Predictors	Past week NSSI engagement (frequency)			Past month NSSI engagement (frequency)			Past year NSSI engagement (frequency)			Lifetime NSSI engagement (frequency)		
	Estimates	CI	*p*	Estimates	CI	*p*	Estimates	CI	*p*	Estimates	CI	*p*
(Intercept)	1.56	−0.09 to 3.21	0.064	3.36	0.96 to 5.75	0.006	10.1	6.12 to 14.08	< 0.001	18.88	14.36 to 23.41	< 0.001
ERSM positive	−0.02	−0.09 to 0.04	0.478	−0.06	−0.15 to 0.03	0.217	−0.2	−0.36 to −0.05	**0.01**	−0.33	−0.50 to −0.16	**< 0.001**
Observations	121			121			121			121		
*R* ^2^/*R* ^2^ adjusted	0.004/−0.004			0.013/0.005			0.054/0.046			0.105/0.098		

*Note:* Bold values indicate *p* 〈 0.05 for all tables.

Abbreviation: ERSM positive, positive emotional responses to social media.

Negative ERSM was positively associated with the presence of NSSI urges across the lifetime, past year, past month, and past week (Table 4).

**TABLE 4:** Results from logistic regression models testing associations between negative emotional responses to social media and presence of NSSI urges.

Predictors	Past week NSSI urge (presence)			Past month NSSI urge (presence)			Past year NSSI urge (presence)			Lifetime NSSI urge (presence)		
	Odds ratios	CI	*p*	Odds ratios	CI	*p*	Odds ratios	CI	*p*	Odds ratios	CI	*p*
(Intercept)	0.05	0.02–0.12	< 0.001	0.06	0.02–0.13	< 0.001	0.09	0.04–0.18	< 0.001	0.1	0.05–0.20	< 0.001
ERSM negative	1.05	1.02–1.08	**< 0.001**	1.06	1.03–1.08	**< 0.001**	1.06	1.03–1.08	**< 0.001**	1.06	1.03–1.08	**< 0.001**
Observations	408			408			408			408		
*R* ^2^ Tjur	0.03			0.043			0.054			0.058		

*Note:* Bold values indicate *p* 〈 0.05 for all tables.

Abbreviation: ERSM negative, negative emotional responses to social media.

Higher negative ERSM was associated with greater probability of lifetime NSSI engagement presence, as well as in the past year, past month, and past week (Table 5). Negative ERSM scores were associated with the frequency of NSSI engagement in the past year, past month, and past week, but not in the lifetime time frame (Table 6).

**TABLE 5:** Results from logistic regression models testing associations between negative emotional responses to social media and presence of NSSI.

Predictors	Past week NSSI engagement (presence)			Past month NSSI engagement (presence)			Past year NSSI engagement (presence)			Lifetime NSSI engagement (presence)		
	Odds ratios	CI	*p*	Odds ratios	CI	*p*	Odds ratios	CI	*p*	Odds ratios	CI	*p*
(Intercept)	0.01	0.00–0.02	< 0.001	0.03	0.01–0.07	< 0.001	0.05	0.02–0.11	< 0.001	0.09	0.04–0.19	< 0.001
ERSM negative	1.09	1.05–1.14	**< 0.001**	1.06	1.03–1.10	**< 0.001**	1.06	1.03–1.09	**< 0.001**	1.05	1.03–1.08	**< 0.001**
Observations	408			408			408			408		
*R* ^2^ Tjur	0.05			0.04			0.054			0.047		

*Note:* Bold values indicate *p* 〈 0.05 for all tables.

Abbreviation: ERSM negative, negative emotional responses to social media.

**TABLE 6:** Results from linear regression models testing associations between negative emotional responses to social media and frequency of NSSI.

Predictors	Past week NSSI engagement (frequency)			Past month NSSI engagement (frequency)			Past year NSSI engagement (frequency)			Lifetime NSSI engagement (frequency)		
	Estimates	CI	*p*	Estimates	CI	*p*	Estimates	CI	*p*	Estimates	CI	*p*
(Intercept)	−1.36	−2.72 to −0.01	0.048	−0.98	−2.98 to 1.02	0.333	1.43	−2.03 to 4.88	0.415	10.4	6.28 to 14.51	< 0.001
ERSM negative	0.07	0.03 to 0.11	**0.001**	0.09	0.03 to 0.15	**0.004**	0.11	0.01 to 0.21	**0.037**	0	−0.12 to 0.13	0.937
Observations	121			121			121			121		
*R* ^2^/*R* ^2^ adjusted	0.096/0.089			0.068/0.060			0.036/0.028			0.000/−0.008		

*Note:* Bold values indicate *p* 〈 0.05 for all tables.

Abbreviation: ERSM negative, negative emotional responses to social media.

## 3 Study 2: Intensive Longitudinal Examination

### 3.1 Methods

#### 3.1.1 Participants

Participants were included from an ongoing study examining pathways linking social media use to teen mental health outcomes. Eligibility criteria included being between the ages of 14–17, in grades 9–12, speaking English fluently, residing in the United States, and using social media. Individuals were required to use Android phones because the larger study's aims collected passive smartphone sensing data related to application use, which was not available in Apple phones due to iOS restrictions. This requirement was not relevant to the aims of the current substudy. Participants were also asked to provide their residential address both to ensure they resided in the US and for delivery of study materials that were used in the larger study and not included in the current substudy, which also served to verify that participants resided in the US. Participants were recruited through flyers and Instagram advertisements. Compensation was provided for up to $210 for completing all study activities required of the larger study and was distributed via Amazon gift cards.

Participants in the current study included 80 adolescents (38.8% White, 85% female). The study was approved by the local university Institutional Review Board.

#### 3.1.2 Procedure

To participate in the study, participants completed an initial eligibility screening questionnaire. Once determined eligible, participants and a parent or legal guardian met with the research team via Zoom to complete consent and assent. Participants then began the 8‐week intensive monitoring period of the study, which included three brief daily ecological momentary assessment surveys.

Participants completed surveys in the morning, afternoon, and evening and indicated their preferred times for the surveys during a study visit. Morning surveys were sent ideally 15 min after waking up, ranging from 6:00 am to 11:00 am on weekdays and 7:00 am to 1:00 pm on weekends. Afternoon surveys were sent randomly between 3:00 pm and 6:00 pm on weekdays and 1:00 pm to 6:00 pm on weekends. Evening surveys were sent ideally 30 min prior to participants' bedtime, ranging from 8:00 pm to 11:00 pm on both weekdays and weekends. Participants were given 60 min to complete morning and afternoon questionnaires and 90 min to complete the evening survey, with reminders given every 30 min. Survey times matched participant time zones. All surveys included safety information and suicide prevention resources.

#### 3.1.3 Measures

##### 3.1.3.1 Emotional Responses to Social Media (ERSM)

A total of 7 items from the ERSM scale (Nesi et al. 2022) were included in the morning, afternoon, and evening daily surveys to measure emotional responses from participants' most recent social media experience. Four items from the negative subscale and 3 items from the positive subscale were included. Responses ranged from 0 (Not at all) to 6 (Extremely). Items were summed for each subscale at each prompt (Hamilton, Dalack, et al. 2024; Hamilton, Untawale, et al. 2024). The full items are detailed in the Supporting Information S1.

##### 3.1.3.2 NSSI Urges (Intensity)

One item in the daily evening surveys assessed NSSI urges (“How intense was your urge to hurt yourself on purpose today without trying to kill yourself (whether you did it or not)?”), with possible responses ranging from 0 (Not at all) to 100 (Extreme) (Hamilton, Dalack, et al. 2024; Hamilton, Untawale, et al. 2024).

##### 3.1.3.3 NSSI Presence (Presence/Absence)

One item in the daily evening surveys assessed whether participants engaged in NSSI during the day (“Today, I hurt myself on purpose but was not trying to kill myself”), with responses coded No = 0 and Yes = 1.

#### 3.1.4 Analytic Plan

Multilevel models examined whether more‐than‐usual positive and negative daily emotional responses to social media are associated with same‐day NSSI urges and engagement. Random intercepts were used to allow individuals to vary in NSSI urges or engagement. Intra‐class correlations were calculated to ensure enough variability at the within‐person level for NSSI urges. Independent predictors of ERSM (positive and negative) used within‐person centering at the prompt level to examine whether deviations (higher or lower levels) from an individual's usual emotional responses over the study period were associated with same‐day NSSI urges and engagement. By doing so, individual variability in emotional responses to social media within each individual was accounted for. Day in the study was used as a covariate to isolate effects at the same‐day level.

### 3.2 Results

The study included a diverse sample, with 49 out of 80 total participants (61.3%) identifying as a racial or ethnic minority and 31 participants (38.8%) identifying as transgender. Additionally, 49 participants (61.3%) identified as a sexual minority. Out of 4480 possible submissions for each prompt, participants completed 2839 morning surveys (63%), 2938 midday surveys (66%), and 3067 evening surveys (68%). See Table 7 for complete demographic breakdown.

**TABLE 7:** Demographic information for Study 2.

	Response, *N* (%) or mean (SD) (*N* = 80)
Age	16.1 (1.0)
Race	
Asian	8 (10.0%)
White	31 (38.8%)
African American or Black	14 (17.5%)
Multiracial	19 (23.8%)
Hispanic/Latino/Latina/Latinx	7 (8.8%)
American Indian/Alaska Native	1 (1.3%)
Sex	
Female	68 (85.0%)
Male	12 (15.0%)
Gender	
Girl/Woman	40 (50.0%)
Boy/Man	16 (20.0%)
Another Gender	24 (30.0%)
Transgender	
No	49 (61.3%)
Yes	31 (38.8%)
Sexual orientation	
Heterosexual	31 (38.8%)
LGBQ+	49 (61.3%)

*Note:* LGBQ+ = acronym encompassing any participants whose sexual orientation is not heterosexual (e.g., gay, lesbian, bisexual).

A total of 40 participants (50.0%) experienced NSSI urges and 17 (21.25%) engaged in NSSI at any point throughout the study. On average, participants endorsed experiencing NSSI urges on 9.93 days during the study period (SD = 12.15) and reported engaging in NSSI on 3.71 days (SD = 3.67).

Average positive ERSM score was 6.29 (SD: 4.76), with a range from 0 to 18. Average negative ERSM score was 4.45 (SD: 4.81), with a range from 0 to 24. Positive and negative ERSM scores were positively correlated (*r* = 0.29, *p* < 0.001).

#### 3.2.1 ERSM and NSSI Primary Results

Intraclass correlation coefficients (ICCs) were calculated to assess the proportion of variance in NSSI urges attributable to between‐person differences but were not calculated for NSSI engagement due to binary responses. ICCs were 0.40 for NSSI urges, suggesting that about half of the variability in NSSI urges can be explained by within‐person differences.

Multilevel models indicated no significant relationship between positive ERSM and NSSI urge intensity (Table 8), although there was a significant relationship between negative ERSM and NSSI urge intensity (Table 9).

**TABLE 8:** Results from multilevel models testing associations between positive emotional responses to social media and NSSI urges and engagement.

Predictors	NSSI urge			NSSI		
	Estimates	CI	*p*	Odds ratios	CI	*p*
(Intercept)	4.84	2.52 to 7.15	< 0.001	0	0.00 to 0.01	< 0.001
ERSM positive	−0.02	−0.16 to 0.12	0.781	1.06	0.97 to 1.16	0.164
Study Day	0	−0.03 to 0.03	0.88	1	0.98 to 1.02	0.915
*Random effects*						
*σ* ^2^	144.89			3.29		
τ00	95.12			12.60		
ICC	0.4			—		
N	80			80		
Observations	3065			3065		
Marginal *R* ^2^/conditional *R* ^2^	0.000/0.396			0.002/0.793		

Abbreviations: ERSM positive, positive emotional responses to social media; ICC, intraclass correlation.

**TABLE 9:** Results from multilevel models testing associations between negative emotional responses to social media and NSSI urges and engagement.

Predictors	NSSI urge			NSSI		
	Estimates	CI	*p*	Odds ratios	CI	*p*
(Intercept)	4.47	2.16 to 6.79	< 0.001	0	0.00 to 0.01	< 0.001
ERSM negative	0.37	0.23 to 0.51	**< 0.001**	1.13	1.04 to 1.24	**0.006**
Study Day	0.01	−0.02 to 0.03	0.693	1	0.98 to 1.02	0.834
*Random effects*						
*σ* ^2^	143.61			3.29		
τ00	95.17			13.23		
ICC	0.4			—		
*N*	80			80		
Observations	3065			3065		
Marginal *R* ^2^/conditional *R* ^2^	0.005/0.402			0.009/0.803		

Abbreviations: ERSM negative, negative emotional responses to social media; ICC intraclass correlation.

Similarly, multilevel models did not demonstrate a significant relationship between positive ERSM and NSSI engagement presence (Table 8), but there was a significant relationship between negative ERSM and NSSI engagement presence (Table 9).

## 4 Discussion

The current study examined adolescents' associations between positive and negative emotional responses to social media and NSSI urges and engagement. To better understand these relationships, a two‐study approach was used. Findings from the cross‐sectional study, Study 1, demonstrated that greater positive emotional responses to social media were associated with lower probability of experiencing past week, month, year, and lifetime NSSI urges, and a lower frequency of engagement in one's lifetime and in the past year. Greater negative emotional responses to social media were associated with higher probability of experiencing both NSSI urges and engagement in the past week, month, year, and lifetime. Greater negative emotional responses to social media were additionally associated with a higher frequency of NSSI engagement in the past week, year, and month. Study 2, which utilized ecological momentary assessment to identify daily associations, found that greater‐than‐usual positive emotional responses to social media were not associated with NSSI urge intensity nor probability of engagement, whereas greater‐than‐usual negative emotional responses to social media were associated with both greater NSSI urge intensity and probability of engagement. Across the two studies, negative responses to social media were consistently associated with greater NSSI engagement and urges. However, positive responses to social media were associated with lower NSSI engagement and urges only in the cross‐sectional study.

Positive emotional responses to social media showed mixed associations with NSSI outcomes. Specifically, positive responses were linked with a lower probability of experiencing NSSI urges across timeframes and a lower frequency of NSSI engagement in the past year and in one's lifetime, but not with a lower probability of NSSI engagement itself. This may be because positive emotional responses to social media could regulate emotional distress, which can potentially buffer against thoughts of NSSI, including urges. It is also possible that additional emotion regulation strategies may be needed to protect against NSSI engagement, but for participants with a history of NSSI, positive emotional responses to social media may protect against further or more frequent engagement. As positive emotional responses to social media were not significantly associated with daily NSSI urge intensity nor presence of engagement, additional coping strategies may be needed to prevent more immediate NSSI engagement. As connectedness is a known protective factor for adolescent suicide and NSSI (Shi et al. 2025), it is possible that social media experiences that foster connection or other positive emotions may play a more cumulative, long‐term buffering role for NSSI. However, due to the cross‐sectional nature of Study 1, it is not possible to infer directionality of these relationships. Therefore, it is also possible that adolescents with lower levels of NSSI in more distant time frames may be having more positive emotions to social media experiences or may be seeking out more positive experiences on social media. Future studies should use a longitudinal design to lend support to the directionality of these constructs. It is also possible that positive and negative emotional responses may interact with one another and have a unique combined association with NSSI‐related outcomes. Of note, positive and negative emotional responses were negatively correlated cross‐sectionally, but were positively correlated in the repeated‐assessments sample, showing that they may represent distinct constructs that may need further assessment. While the current study examined these constructs separately, future studies may examine the interaction between positive and negative responses in relation to NSSI.

These findings also contrast with past literature suggesting that positive affect from social media experiences may reflect adolescents' reliance on social media for the peer support and self‐expression that they lack from in‐person experiences (Nesi et al. 2022), such that there is an association between greater NSSI engagement and higher levels of online social support (Tang et al. 2021). While positive emotional responses to online connection may account for a lower probability of NSSI, other positive experiences adolescents have on social media (e.g., viewing positive content, identity exploration) may also elicit positive emotions and sustained affect. Nonetheless, the observed association between positive emotional responses and lower NSSI urges and engagement aligns with findings that momentary positive affect is inversely related to NSSI urges (Kiekens et al. 2020) and that greater positive emotions predict lower NSSI risk (Kranzler et al. 2016). Future research may differentiate between types of positive responses to specific online experiences to clarify how various experiences may elicit different emotions and thus result in different mental health outcomes.

Across both study approaches, greater negative emotional responses to social media were associated with a higher likelihood of NSSI urges and engagement, stronger NSSI urge intensity, and higher frequency of NSSI engagement. Cross‐sectionally, greater negative emotional responses to social media were associated with greater probability of experiencing NSSI urges, engagement, and a greater frequency of engagement—though not lifetime frequency of engagement. At the daily level, participants who experienced more negative social media responses than usual also reported more intense NSSI urges and were more likely to engage in NSSI that day. These findings align with current models of NSSI, which theorize that NSSI is commonly used to regulate distressing emotional states (Houben et al. 2017). Thus, adolescents might engage in NSSI following same‐day or recent distressing emotions responding to negative social media experiences as a way to cope.

Alternatively, individuals already experiencing NSSI urges or engaging in NSSI may be more likely to seek negative social media experiences or respond more negatively to online experiences. Of note, the present analyses also do not clarify whether the observed associations in the intensive‐longitudinal portion of the study differ among individuals with or without prior NSSI history. Given that 50% of participants in the intensive‐longitudinal portion of the study experienced NSSI urges, it is possible that observed associations were driven by this subgroup, and results may not extend to individuals who have never experienced any NSSI or NSSI urge. Future work should assess whether these daily associations differ depending on prior NSSI history.

Our findings may be useful in creating targeted interventions for NSSI, which may focus on regulating negative emotions in response to distressing social media experiences. Although the present study focused on subjective emotional responses to social media, future work may also consider examining objective social media experiences, such as the type of content that adolescents interact with. For instance, viewing NSSI‐related photos online may be associated with NSSI behavior, and simultaneously, mental imagery of NSSI may also precede engagement (Baker and Lewis 2013; Ji et al. 2024; Lawrence et al. 2023). Thus, future research may consider examining links between viewing NSSI images online, NSSI mental imagery, and subsequent engagement.

The current study contained several strengths, such as utilizing both a cross‐sectional and longitudinal design and including a diverse sample. Nevertheless, certain limitations should be taken into account. The study did not compare participant responses across different time points, and the nature of cross‐sectional research prevents temporal interpretations of data. Future research would benefit from comparing emotional responses to social media with NSSI urges and engagement at subsequent time points. By doing so, evidence may lend support to the predictive nature of emotional responses to social media in NSSI and may elucidate whether positive and negative emotional responses to social media have an immediate or prolonged effect on NSSI.

The current study is also limited by our assessment of NSSI, which was measured differently across the cross‐sectional and intensive longitudinal components due to differences in data availability. This may prevent direct comparisons of results between study components. Future studies should include NSSI variables (presence of urges, urge intensity, presence of engagement, and engagement frequency) at both cross‐sectional and intensive longitudinal levels for more direct comparisons. Furthermore, NSSI was only assessed once per day in the momentary component of the study to ease participant burden, whereas emotional responses to social media were assessed three times per day. Again, this makes it difficult to draw conclusions about whether emotional responses to social media preceded or followed NSSI urges and engagement.

Additionally, items asking about NSSI urges and engagement in the past week, month, year, and lifetime in the cross‐sectional component of the study were assessed retroactively. This may introduce recall bias, as adolescents may misremember their history of NSSI or may have perceived past experiences differently than they occurred. Future research may consider assessing NSSI more in‐depth at multiple time points each day in an ecological momentary assessment design to clarify real‐time dynamics, reduce reliance on retroactive reporting, and further understand the relationship between emotional responses to social media and NSSI. Finally, although the diverse sample may extend literature by including underrepresented groups, a majority sexual minority sample (61%) may not be generalizable more broadly to all adolescents.

The current study has key implications for adolescent social media use. Given that negative emotional responses to social media were a strong predictor of NSSI urges and engagement, it is possible that adolescents' engagement with social media may rely on their emotional responses rather than screen time. These findings add to growing evidence that other objective measures of screen time may not accurately predict mental health outcomes (Hamilton, Dalack, et al. 2024; Hamilton, Untawale, et al. 2024). For interventions targeting NSSI in adolescents, caregivers and clinicians may consider focusing on improving emotion regulation, with the goal of decreasing the intensity of negative emotional responses to social media, rather than limiting social media use itself.

## 5 Conclusion

As social media becomes an increasingly prevalent factor in adolescent mental health outcomes, it is important to assess youth's subjective emotional responses to social media. The current study leveraged two distinct datasets to examine associations between positive and negative emotional responses to social media and the presence/absence of NSSI urges and engagement, intensity of NSSI urges, and frequency of NSSI engagement. Findings highlight the potential double‐edged nature of emotional responses to social media, as positive responses were associated with a lower probability of experiencing NSSI urges in the past week, month, year, and lifetime, as well as frequency of engagement in the past year and lifetime. Negative responses to social media were associated with a greater probability of experiencing NSSI urges and engagement in the past week, month, year, and lifetime, and frequency of engagement in the past week, month, and year. Given that positive responses to social media were not associated with NSSI urge intensity nor engagement presence at the daily level, whereas greater‐than‐usual negative responses to social media were associated with more intense NSSI urges and greater probability of engagement at the daily level, negative responses to social media may be more proximally linked with NSSI than its potential protective effects. Together, these findings suggest that interventions reducing adolescent NSSI may benefit from addressing ways to cope with negative social media experiences and perhaps also promoting more positive social media experiences or behaviors. Future research should utilize a longitudinal design to investigate whether emotional responses to social media precede or follow NSSI urges and engagement, which may reveal emotional responses to social media as a potential protective or risk factor for adolescent NSSI.

## Funding

This work was supported by the NIMH K01MH121584 and R21MH135493; PI: JLH, and F31MH136815 (PI: MJD).

## Ethics Statement

All procedures were performed in compliance with the Rutgers University Institutional Review Board (Reference number: Pro2020002200, Date of approval: 9/17/21; Reference number: Pro2022001324, Date of approval: 1/24/2023).

## Consent

Privacy rights of human subjects have been observed. Informed consent was received from all participants.

## Conflicts of Interest

The authors declare no conflicts of interest.

## Supporting information


**Data S1:** List of items for the emotional responses to social media cross‐sectional measure used in Study 1 and the ecological momentary assessment version used in Study 2.

## Data Availability

The data that support the findings of this study are available from the corresponding author upon reasonable request.
