# Supplementary material for: Emotional Responses to Social Media and Non‐Suicidal Self‐Injury Among Adolescents
Source: Suicide Life Threat Behav. 2026 Jun 25;56(3):e70122. doi: 10.1111/sltb.70122 (PMC13296832; doi:10.1111/sltb.70122)
Supplement: Supplementary file 1 — Data S1: List of items for the emotional responses to social media cross‐sectional measure used in Study 1 and the ecological momentary assessment version used in Study 2. [file SLTB-56-0-s001.docx]

**Study 1 ERSM Items**

When you use social media, how often do you...

1. Feel creative because of something you expressed, posted, or shared?
2. Feel proud of yourself because of something you expressed, posted, or shared
3. Feel hurt by a negative interaction (e.g., comment, post, DM) with someone?
4. Feel more connected to your friends
5. Feel left out or excluded by friends or people you know?
6. Feel supported and encouraged by friends or people you know?
7. Feel disappointed about not getting enough responses (likes, comments, views, or shares)?
8. Feel pressure to show the best version of yourself?
9. Feel happy because of a positive interaction (e.g., comment, post, DM) with someone?
10. Feel like other people are doing better than you?
11. Feel worried that you are missing out on things?
12. Feel like you are enjoying yourself and having fun?
13. Feel anxious when you are waiting for someone to respond?
14. Feel pressure to be available to others (e.g., respond right away)?
15. Feel stressed or anxious about something you see online?
16. Feel like you are accepted for who you are?
17. Feel happy or good about yourself compared to other people your age (such as in appearance, accomplishments, relationships, or other things)?
18. Feel excited or happy that you got likes, comments, views, or shares on your posts?
19. Feel uncertain or nervous about how others will respond to the things you post on social media?
20. Feel out of control about how much time you spend on social media?
21. Feel frustrated by other people posting things to appear a certain way on social media?
22. Feel overwhelmed by the number of things you need to look at or respond to on social media?
23. Feel that social media has a negative impact on your mood?
24. Feel that social media has a positive impact on your mood?

Each item is rated on a Likert scale from 0 (never) to 4 (always).

Positive subscale: Items 1, 2, 4, 6, 9, 12, 16, 17, 18, 24

Negative subscale: Items 3, 5, 7, 8, 10, 11, 13, 14, 15, 19, 20, 21, 22, 23

**Study 2 EMA ERSM Items**

Thinking about the last time you used social media, how much did you

feel…

1. That you aren’t as good (e.g., attractive/accomplished/etc) or popular as other people?
2. Worried that you were missing out on things?
3. Nervous to post something because of what other people might think or say?
4. Sad or hurt because of a negative interaction (e.g., comment, post, DM) with someone else?
5. Inspired by other people on something you saw?
6. Supported or encouraged by others?
7. Happy or excited because of a positive interaction (e.g., comment, post, DM) with someone else?

Each item is rated on a Likert scale from 0 (not at all) to 6 (extremely).

Positive subscale: Items 5, 6, 7

Negative subscale: Items 1, 2, 3, 4
